# Emergency department CT examinations demonstrate no evidence of early viral circulation at the start of the COVID-19 pandemic—a multicentre epidemiological study

**DOI:** 10.1186/s13244-023-01590-8

**Published:** 2024-01-17

**Authors:** Amandine Crombé, Claire Dupont, François Casalonga, Mylène Seux, Nicolas Favard, Agnès Coulon, Thomas Jurkovic, Hubert Nivet, Guillaume Gorincour

**Affiliations:** 1 IMADIS, Lyon, France; 2 SARCOTARGET Team, BRIC INSERM U1312 - Bordeaux University, Bordeaux, F-33000 France; 3 Department of Radiology, Pellegrin University Hospital, Bordeaux, France; 4 IMASUD, Toulon, France; 5 Imagerie Médicale du Mâconnais, Mâcon, France; 6 Centre Léon Berard, Lyon, France; 7 Centre Aquitain d’Imagerie Médicale, Mérignac, France; 8 Clinique Bouchard, ELSAN, Marseille, France

**Keywords:** Computed tomography, Time series analysis, Coronavirus disease 2019, SARS-CoV-2, Report text mining

## Abstract

**Background:**

Biological studies suggested that the COVID-19 outbreak in France occurred before the first official diagnosis on January 24, 2020. We investigated this controversial topic using a large collection of chest CTs performed throughout French emergency departments within 6 months before the 1st lockdown.

**Results:**

Overall, 49,311 consecutive patients (median age: 60 years, 23,636/49,311 [47.9%] women) with available chest CT images and reports from 61 emergency departments between September 1, 2020, and March 16, 2020 (day before the 1st French lockdown), were retrospectively included in this multicentre study. In the macroscopic analysis of reports automatically (labelled for presence of ground glass opacities [GGOs], reticulations, and bilateral and subpleural abnormalities), we found a significant breakpoint on February 17, 2020, for the weekly time series with 1, 2 and ≥ 3 of these 4 radiological features, with 146/49,311 (0.3%) patients showing bilateral abnormalities and ground glass opacities (GGOs) from that day. According to radiologists, 22/146 (15.1%) CT images showed typical characteristics of COVID-19, including 4/146 (2.7%) before February 2020. According to hospital records, one patient remained without microbial diagnosis, two patients had proven influenza A and one patient had concomitant influenza A and mycoplasma infection.

**Conclusion:**

These results suggest that SARS-CoV-2 was not circulating in the areas covered by the 61 emergency departments involved in our study before the official beginning of the COVID-19 outbreak in France. In emergency patients, the strong resemblance among mycoplasma, influenza A and SARS-CoV-2 lung infections on chest CT and the nonspecificity of CT patterns in low prevalence periods is stressed.

**Critical relevance statement:**

We proposed here an innovative approach to revisit a controversial ‘real’ start of the COVID-19 pandemic in France based on (1) a population-level approach combining text mining, time series analysis and an epidemiological dataset and (2) a patient-level approach with careful retrospective reading of chest CT scans complemented by analysis of samples performed contemporarily to the chest CT. We showed no evidence that SARS-CoV-2 was actively circulating in France before February 2020.

**Graphical Abstract:**

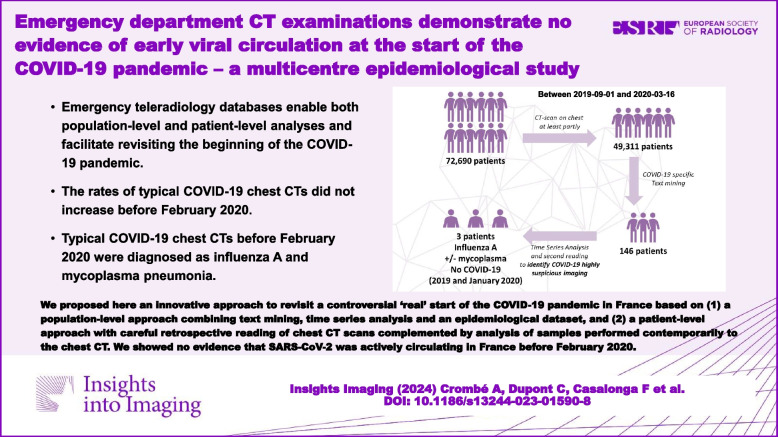

**Supplementary Information:**

The online version contains supplementary material available at 10.1186/s13244-023-01590-8.

## Key points

1. Emergency teleradiology databases enable both population-level and patient-level analyses and facilitate revisiting the beginning of the COVID-19 pandemic.

2. The rates of typical COVID-19 chest CTs did not increase before February 2020.

3. Typical COVID-19 chest CTs before February 2020 were diagnosed as influenza A and mycoplasma pneumonia.

**Supplementary Information:**

The online version contains supplementary material available at 10.1186/s13244-023-01590-8.

## Background

Coronavirus disease 2019 (COVID-19) has become endemic worldwide. Latest radiological researches on COVID-19 have shown that the omicron strain and especially the newer omicron variant (BA.5) of the severe acute respiratory syndrome coronavirus 2 (SARS-CoV-2) led to higher rates of transmission and decreased severity of infection, subsequently acting as an endemic viral infection [1–3]. To better anticipate the next pandemic, health agencies investigated the first weeks of the COVID-19 outbreak.

The first French patients with biologically proven SARS-CoV-2 infection were diagnosed on January 24, 2020 [4, 5]. Spreading of the disease seemed contained during February but rapidly increased in March, from 37 patients with positive RT-PCR on March 1, 2020, to 4611 patients on March 28 [4, 5]. This number is probably underestimated because tests were developed in late January by the French National Reference Center and available in hospitals from March. Consequently, controversies arose about the start of the COVID-19 pandemic. Retrospective RT-PCRs performed on stored samples from patients with flu-like symptoms in intensive care units (ICUs) in the Paris region in December 2019 and January 2020 identified one positive patient [6]. Carrat et al. identified 11 positive patients from the CONSTANCES cohort between November 2019 and January 2020 [7]. Overall, these studies suggested that SARS-CoV-2 was already circulating in France weeks before the first official cases.

Teleradiology uses interoperable information tools for imaging requests, reports and storage with the ability to rapidly collect data from all the centres working with a given partner. IMADIS is fully dedicated to remote interpretation of emergency imaging throughout France. We showed that the number of chest CTs performed in a workflow devoted to COVID-19 was strongly correlated with the number of patients hospitalised for proven COVID-19 infection, ICU admission and COVID-19-related deaths [8]. Moreover, several studies have stressed the diagnostic accuracy of chest CT for COVID-19 and its ability to assess disease extension [9–12], which has been formalised into diagnostic and severity scores promoted by radiological societies since April 2020 [1, 13, 14]. According to the French Society of Radiology–French Society of Thoracic Imaging (SFR-SIT) diagnostic score, a typical chest CT had an accuracy of 83–87.9% and a compatible or typical chest CT had an accuracy of 84–88.1% to diagnose positive RT-PCR for SARS-CoV-2 [9]. Integrating semantic radiological features into predictive models could improve those performances [15, 16].

Consequently, given the accuracy of chest CT to diagnose COVID-19, our goal was to complement the biological investigation from an imaging perspective [6, 7]. Using a large collection of reports of chest CTs performed throughout France during the months before the official outbreak, our aim was to investigate whether typical COVID-19 chest CTs were already prevalent and whether biological analyses could confirm the radiological suspicions.

## Materials and methods

### Study design and data collection

This multicentre observational retrospective study was approved by the SFR institutional review board (CRM-2103–147).

**Fig. 1:**
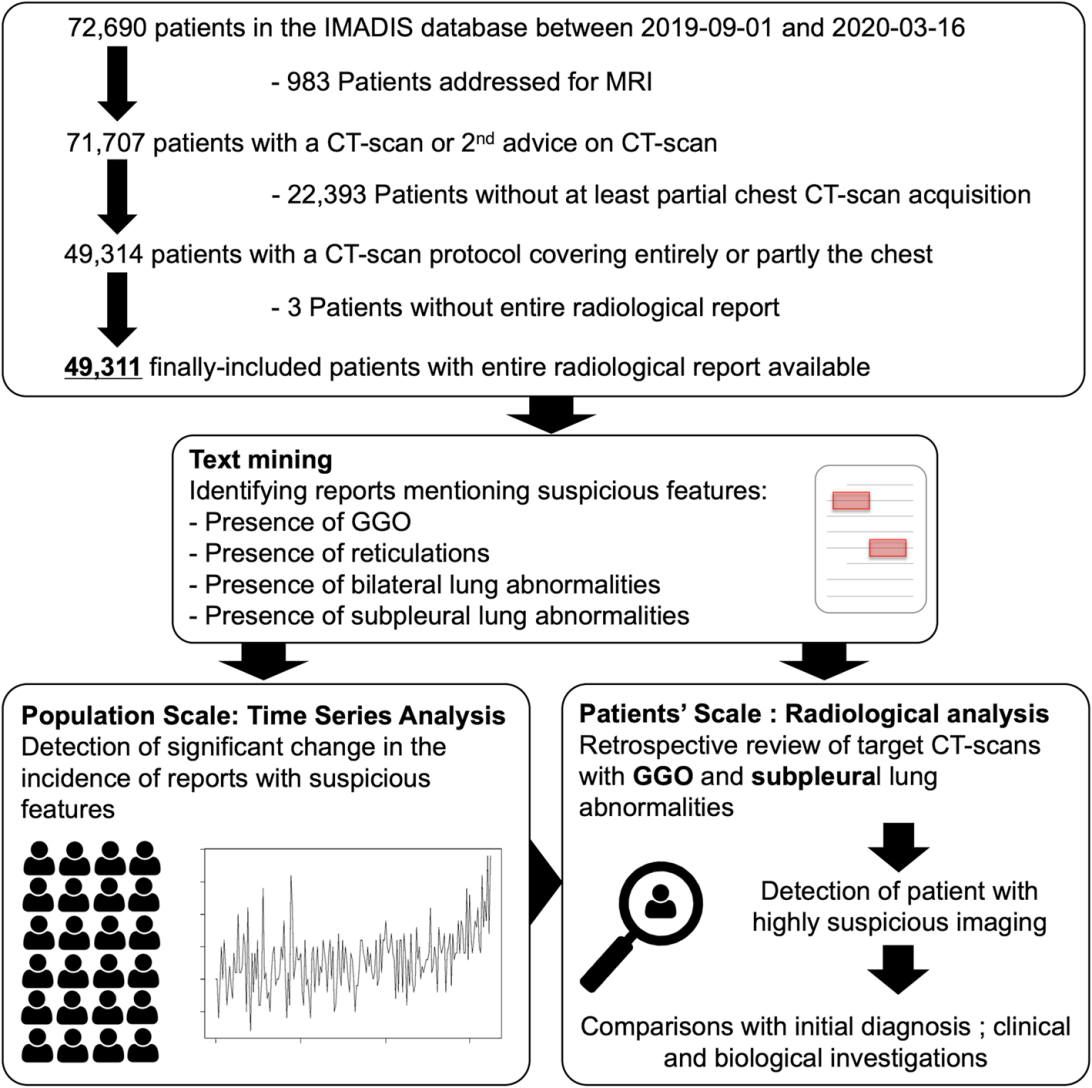
Study flowchart and workflow. Abbreviations: GGO, ground glass opacities

We included all consecutive patients with available radiological reports who underwent CT scans covering the entire or partial chest between September 1, 2019, and March 16, 2020 (day before the 1st lockdown), in the emergency workflow of 61 French partner hospitals of IMADIS teleradiology. Figure 1 shows the flow chart.

IMADIS teleradiology is a French medical company dedicated to the interpretation of medical imaging for private and public emergency departments throughout France. The teleradiology interpretation protocol met the French recommendations for teleradiology practice. Requests by emergency physicians were received from partner hospitals at our teleradiology centres (Lyon, Bordeaux and Marseille, during the study period), using teleradiology software (ITIS, Deeplink Medical, Lyon, France). The images were securely transferred over a virtual private network to a local picture archiving and communication system for interpretation (Carestream Health 12, Rochester, NY, USA). Images were then interpreted by one of the 167 board-certified radiologists working at IMADIS during the study period. Those radiologists had various backgrounds but at least 2.5 years of experience in emergency imaging. They analysed the imaging in dedicated emergency reading rooms in the teleradiology centres during their on-call duty. The CT protocols were automatically and prospectively encoded in the IMADIS database, which enabled to filter the observations entirely including the chest, and those partly covering the chest, namely, the dorsal spine, the abdomen-pelvis (bottom part of the chest) and CT angiography of the supra-aortic trunks (upper part of the chest).

**Fig. 2:**
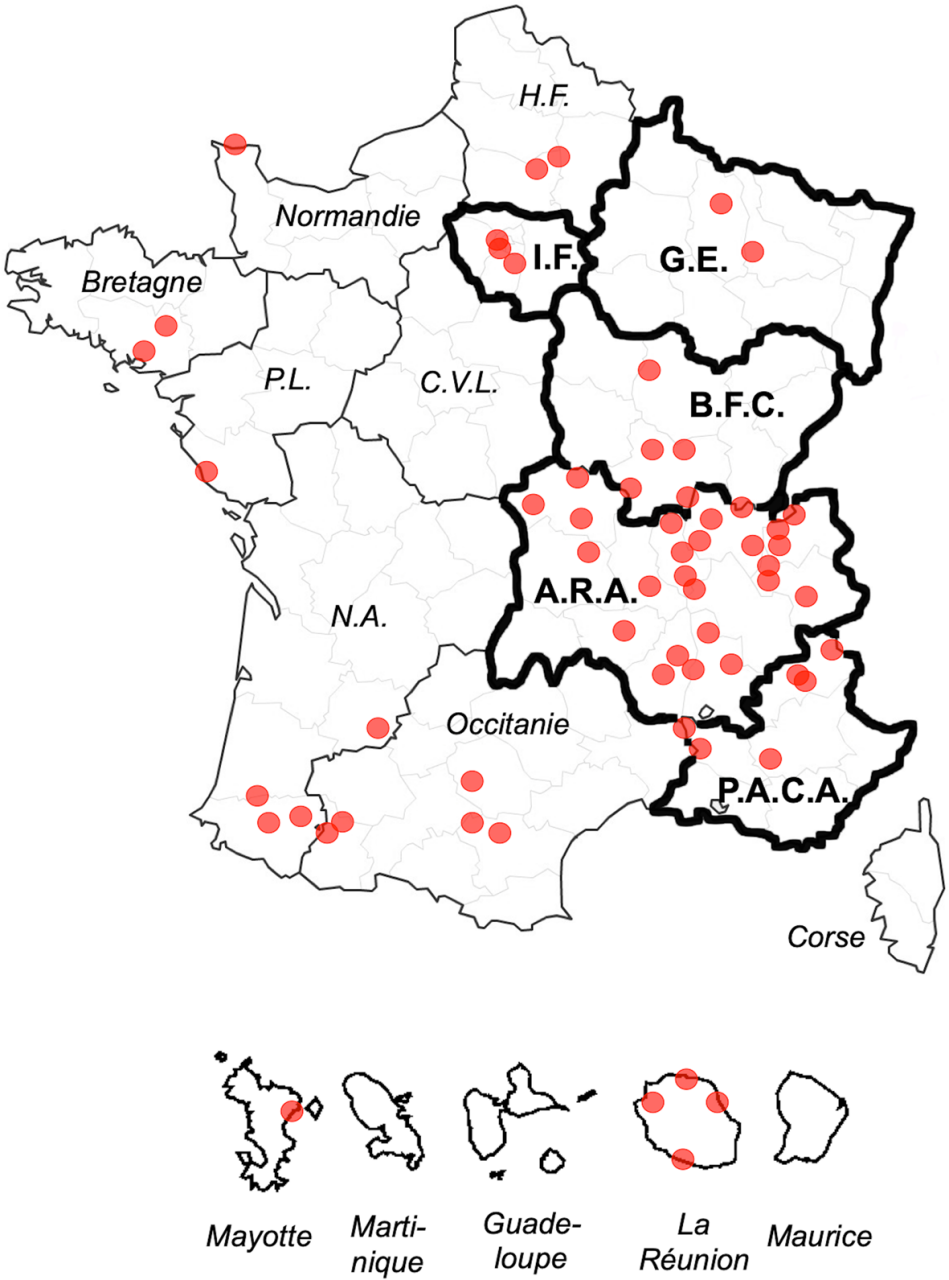
Location of the 61 IMADIS partner centres involved in the study. The centres are indicated with red dots. The great regions where the SARS-CoV-2 virus initially spread are highlighted with thick borders and their names are in bold. Abbreviations: A.R.A., Auvergne Rhône Alpes; B.F.C., Bourgogne Franche Comté; C.V.L., Centre Val de Loire; G.E., Grand Est; H.F., Haut de France; I.F., Ile de France; N.A., Nouvelle Aquitaine; P.A.C.A., Provence Alpes Côte d’Azur; P.L., Pays de la Loire

Data collection consisted in patients’ age, sex, date of CT interpretation, imaging protocol (coverage and use of contrast medium), location of the partner department and whether it was located in a French region where the COVID-19 initially spread (i.e. ‘Grand-Est’, ‘Ile-de-France’, ‘Bourgogne-Franche-Comté’, ‘Auvergne-Rhône-Alpes’, or ‘Provence-Alpes-Côte-d’Azur’, which is also shown on Fig. 2) [5]. The annual number of visits to these centres and the number of inhabitants in the areas covered by these centres were estimated according to the data provided by each hospital.

We also collected the number of COVID-19 patients during the study period from the epidemiological dataset available at *data.gouv.fr*, an open-source platform promoted by French authorities and ‘Santé Publique France’ (SPF dataset) [5]. It must be noted that the SPF dataset and the IMADIS radiological dataset were independent.

### Annotating raw texts

First, we annotated each radiological report using text mining to identify those reports where radiologists noted the presence of ground glass opacities (GGOs), intralobular reticulations, or bilateral and subpleural lung abnormalities in the results and/or conclusion sections. Next, the texts were imported into R (v4.1.0, Vienna, Austria) and preprocessed using the ‘stringr’ package (github.com/tidyverse/stringr, v1.5.0). French accents were removed, and letters were converted to lowercase.

To detect GGOs in textual reports, we first captured the entire sentences containing the French for ‘GGO’ in singular or plural form, including the most common spelling mistakes. We identified whether negative formulations were used preceding GGO in captured sentences by looking for the presence of French equivalents for ‘no’, ‘not’, ‘neither’, ‘without’ or ‘absence of’. If no negative formulation was found, reports were labelled ‘GGO positive’ (and ‘negative’ otherwise).

The same approach was used for ‘reticulation’ to identify ‘reticulation positive’ reports.

Regarding bilateral abnormalities, we captured sentences containing the word ‘bilateral’. Since bilateral abnormal findings can be found outside the chest that have no link to COVID-19, we identified sentences in which ‘bilateral’ was accompanied by the French equivalents for ‘reticulation’, ‘GGO’, ‘fibrosis’, ‘fibrotic’, ‘consolidation’, ‘band’, ‘opacities’, ‘attenuation’ or ‘honeycomb’. We removed sentences with negative formulations before ‘bilateral’. The reports matching these constraints were labelled ‘bilateral positive’.

Regarding subpleural abnormalities, the same approach was used as for ‘bilateral’ abnormalities, except that we captured sentences containing French equivalents for the following variations: ‘sub pleura’, subpleura’, or ‘sub-pleura’. Matching reports were labelled ‘subpleural positive’.

### Quality control of the annotations

To verify the accuracy of these algorithms, we performed a quality control. For each of the four radiological variables, we randomly sampled 100 distinct reports from the entire corpus of reports (50/100 [50%] with positive labels and 50/100 [50%] with negative labels). These 100 reports were carefully read, blinded to the labels from the algorithms, by one senior radiologist (A.Cr., with 5 years of experience in emergency radiology after board certification) in order to provide a reference for the four radiological variables and to confirm that the error rate of the algorithms was < 10% (i.e. accuracy ≥ 90%). In case of doubtful depiction in the sentences of the reports, the radiologist had access to the CT images.

We also calculated the sensitivity, specificity, negative predicted value (NPV), positive predicted value (PPV) and area under the ROC curve (AUROC) with 95% confidence interval (CI) of the four annotation algorithms.

Afterwards, we created a new variable named ‘number of positive features’, which ranged from 0 to 4 depending on the number of labels for ‘positive GGO’, ‘positive reticulation’, ‘positive bilateral’ and ‘positive subpleural’.

### Identifying target CT scans

The next step was to identify CT scans to be reviewed by expert radiologists to detect those highly suspicious for COVID-19. As the presence of subpleural GGOs was constantly depicted as specific, we filtered observations with both ‘positive GGO’ and ‘positive subpleural’ labels and named them ‘target CT scans’ (published PPV = 0.81) [9].

### Radiological analysis of target CT scans

**Fig. 3:**
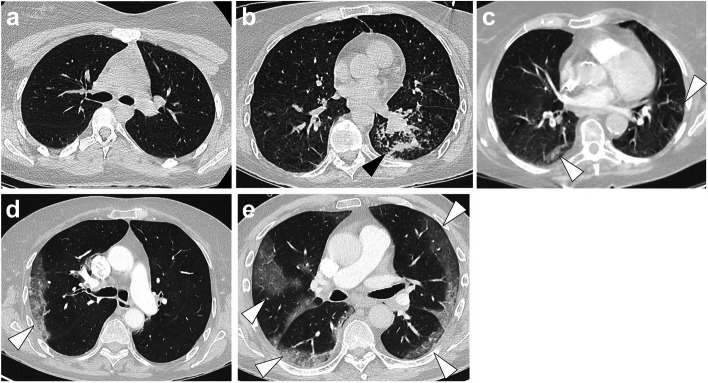
French ‘*Société Française de Radiologie*’ and ‘*Société Française d’Imagerie Thoracique*’ adapted classifications for the radiological findings in the setting of a suspicion of coronavirus disease 2019 (COVID-19), or SFR-SIT diagnostic score, categorised as the following: **a** I: no pathologic findings; **b** II: non-SARS-CoV-2 infections (herein, bacterial bronchopneumonitis, black arrowheads); **c** III: indeterminate (herein, subtle subpleural condensations, white arrowhead); **d** IV: pathologic findings compatible with COVID-19 (herein, a unique subpleural area of ground glass opacity, white arrowhead); **e** V: pathologic findings typical for COVID-19 lung disease (i.e. multiple ground glass opacities seen in ≥ 2 lobes, with a subpleural predominant locations, possibly associated with band-like condensations and reversible fibrosis). All CT images are in axial plane and lung kernel

Seven radiologists reviewed the target CT scans: C.D., A.Co., H.N., G.G., F.C., N.F. and T.J., with 1, 5, 3, 12, 2, 7 and 6 years of experience as senior radiologists in emergency imaging, respectively. Additionally, A.Co., H.N., F.C., N.F. and T.J. had expertise in chest imaging with at least 2 years of specialisation in thoracic imaging department from French University Hospitals after their board certification. CT scans were randomly distributed so that three distinct radiologists analysed each scan. They reported the following radiological features (all binary and categorised as ‘yes’ or ‘no’) blinded to initial reports and to other radiologists’ results: (1) GGO; (2) band-like consolidation; (3) intralobular reticulations; (4) GGO as the predominant pattern; (5) subpleural area as the predominant location of abnormal findings; (6) abnormal findings affecting ≥ 2 lobes; (7) bronchitis syndrome; (8) SFR-SIT diagnostic score (categorised as I: no pathologic findings, II: non-SARS-CoV-2 infections, III: indeterminate, IV: findings compatible with COVID-19, V: findings typical for COVID-19; Fig. 3) [1]. For each binary radiological variable (1–7), the consensus result corresponded to the most frequent finding across the 3 radiologists.

The radiologists were also asked to propose an alternative compatible diagnosis, if possible.

### Clinical and biological investigations in patients with high suspicion of COVID-19

Patients were considered to have highly suspicious chest CTs if the reviewing radiologists classified the CT scan as at least SFR-IV, including at least two SFR-V scores (i.e. SFR-V-V-V and SFR-IV-V-V). At the time of our analysis, most follow-up data were available in our shared information system, enabling us to obtain results from complementary serology or RT-PCR.

### Algorithmic predictions

Based on the analyses of the 7 radiologists, we computed the probability of positive SARS-COV-2 RT-PCR according to two publicly available algorithms, one relying on a classification and regression tree (CART) and the second on stepwise logistic regression (Step-LR) [16]. Details regarding the models are provided in Supplementary Data S1.

### Statistical analysis

Statistical analysis was performed using R (v4.1.0, Vienna, Austria). All tests were two-tailed. A *p*-value < 0.05 was deemed significant.

#### Time series analysis

For each week, we calculated (i) the total number of CT scans interpreted, (ii) the number of CT scans with 1, 2, 3 and 4 positive features identified with text mining and (iii) the number of target CT scans. This data frame was converted to a time series using the ‘xts’ package (github.com/joshuaulrich/xts, v0.12.1). We then applied the breakpoints function from the ‘strucchange’ package to investigate whether and when significant structural changes and breaks occurred during the study period (github.com/cran/strucchange, v1.5–2). This function uses a dynamic programming algorithm and identifies the optimal number of breakpoints in a time series (considered piecewise linear models) that minimises the residual sum of squares (RSS) and the number of parameters in the model according to the Bayesian information criterion (BIC) [17].

Moreover, we compared the age, sex, locations, radiological characteristics and the number of positive radiological features in patients from September 2019 to November 2019 (included) and in patients from December 2019 to February 2020 (i.e. once the COVID-19 pandemic started). We used either chi-square tests for categorical variable or unpaired *t*-test or Mann–Whitney test for numeric variables depending on D’Agostino kurtosis normality test (which applied for dataset with more than 5000 observations, contrarily to classical Shapiro–Wilk normality test). Patients included in March 2020 were excluded as the SARS-CoV-2 was known to actively spread in France at that time [5].

#### Interobserver reproducibility of radiological analysis and algorithm predictions in target CT scans

Since multiple readers analysed different subsets of target CT scans, we used Krippendorff’s alpha (α_K_) from the ‘irr’ package (github.com/staudlex/irr, v0.84.1) [18]. The 95%CIs were computed using bootstrapping on 1000 replicates of the population.

#### Agreement between the initial and second readings of the target CT scans

We calculated the percentage of agreement and Kappa index between the initial prospective CT scan report and the second retrospective readings performed for the presence of GGOs and reticulations.

## Results

### Characteristics of the cohorts and text annotations

Of the 72,690 patients in the IMADIS database during the study period (addressed for MRI or CT scans), 49,311 patients from 61 partner centres were finally included and their CT scans were interpreted by 167 radiologists (Fig. 1). The median patient age was 60 years (range: 0–100). There were 23,636/49,311 (47.9%) female patients (Table 1).

**Table 1:** Characteristics of the study population and of patients with a report mentioning the presence of ground glass opacities and subpleural abnormal findings on chest CT scan (i.e. ‘target reports’)

**Characteristics**	**All patients** **(*n *= 49,311)**	**Target reports (*n *= 146)**
**Sex**		
Women	23,636/49,311 (47.9%)	56/146 (38.4%)
Men	25,675/49,311 (52.1%)	90/146 (61.6%)
**Age (years)**		
Mean ± SD	57.7 ± 22.7	64.3 ± 19.7
Median (range)	59.9 (0–100.1)	67.1 (15.3–95.7)
**Contrast medium injection**		
No	11,607/49,311 (23.5%)	47/146 (32.2%)
Yes	37,704/49,311 (76.5%)	99/146 (67.8%)
**Coverage of the chest**		
Entirely	16,708/49,311 (33.9%)	137/146 (93.8%)
Partly (top)	10,138/49,311 (20.6%)	3/146 (2.1%)
Partly (bottom)	20,531/49,311 (41.6%)	6/146 (4.1%)
Partly (dorsal spine)	1934/49,311 (3.9%)	0/146 (0%)
**No. of positive text features**		
0	46,207/49,311 (93.7%)	0/146 (0%)
1	2539/49,311 (5.1%)	0/146 (0%)
2	494/49,311 (1%)	97/146 (66.4%)
3	65/49,311 (0.1%)	43/146 (29.5%)
4	6/49,311 (0.01%)	6/146 (4.1%)
**Presence of GGO**		
Positive	1721/49,311 (3.5%)	146/146 (100%)
**Presence of intralobular reticulation**		
Positive	263/49,311 (0.5%)	33/146 (22.6%)
**Presence of bilateral lung abnormalities**		
Positive	1324/49,311 (2.7%)	22/146 (15.1%)
**Presence of subpleural lung abnormalities**		
Positive	438/49,311 (0.9%)	146/146 (100%)

Data are numbers of patients with percentages in parentheses except for age

*Abbreviations*: *GGO* ground glass opacities, *SD* standard deviation

Supplementary Data S2 shows the different CT scanners used for the acquisition. Regarding partner centres, 42/61 (68.9%) were located in the French regions where the outbreak started (Fig. 2), which represented 31,793/49,311 (64.5%) of all included examinations. Those centres represented about 2,159,515 annual patients’ visits per year and covered a population basin of 5,744,088 inhabitants.

**Fig. 4:**
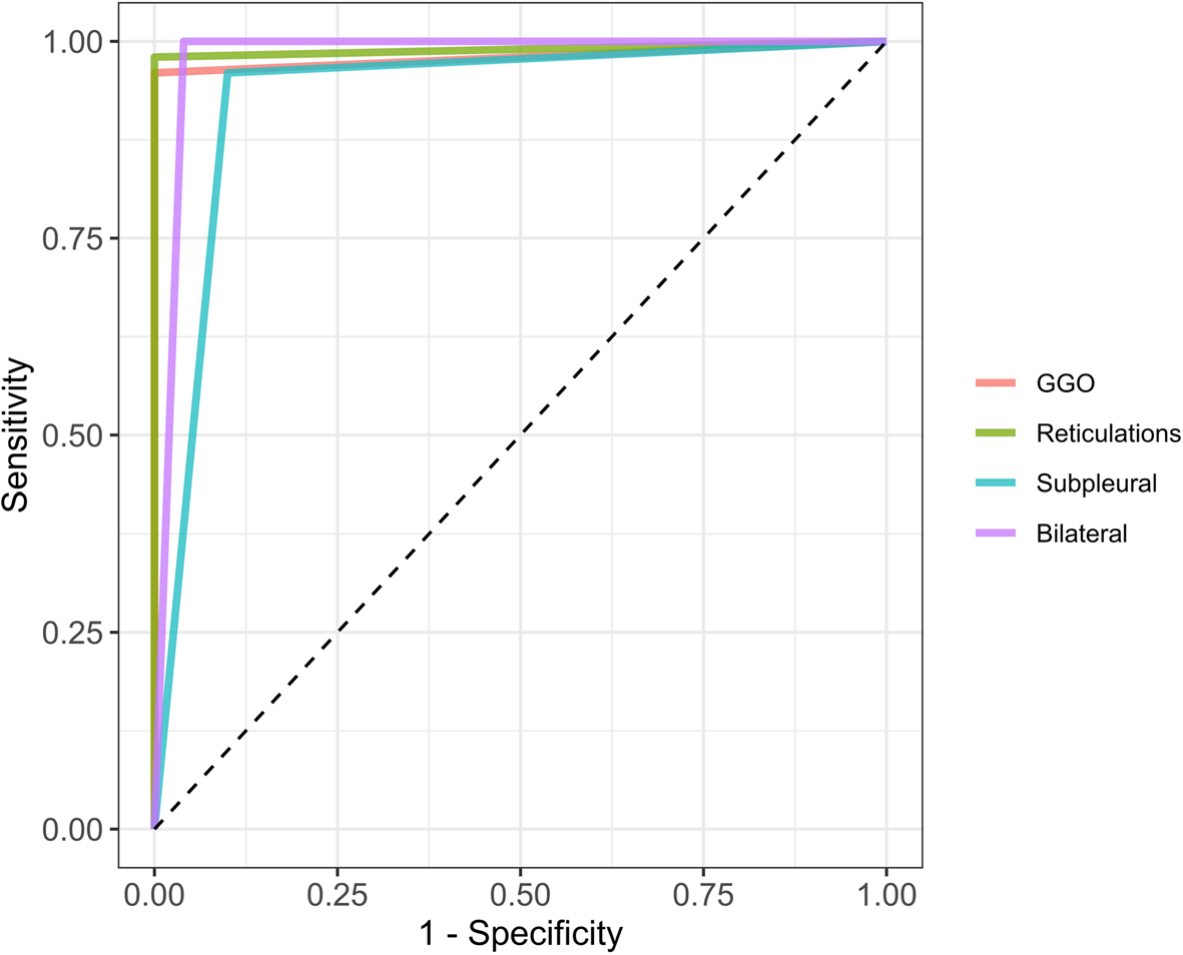
ROC curves of the four rule-based algorithms for automatic annotations of the reports. Abbreviations: GGO, ground glass opacities

The performances of the rule-based algorithms for automatically annotating the reports are given in Table 2. Their accuracy ranged from 93% (95%CI = 86.1–97.1%) for the ‘subpleural’ labelling to 99% (95%CI = 94.6–100%) for ‘reticulation’ labelling. Similarly, their AUROC ranged from 0.93 (95%CI = 0.89–0.98) for ‘subpleural’ labelling to 0.99 (95%CI = 0.97–1) for ‘reticulations (Fig. 4).

**Table 2:** Performances of the rule-based models developed to automatically annotate the texts of the radiological reports

**Variable investigated with text mining**	**Accuracy**	**Error rate**	**Sensitivity**	**Specificity**	**PPV**	**NPV**	**AUROC**
GGO positive	98 (93–99.8)	2%	96 (86.3–99.5)	100 (92.9–100)	93.9 (83.6–97.2)	97.3 (86.6–99.1)	0.98 (0.95–1)
Reticulation positive	99 (94.6–100)	1%	98 (89.4–99.9)	100 (92.9–100)	95.6 (85.3–98.2)	97.4 (86.8–99.1)	0.99 (0.97–1)
Bilateral positive	98 (93–99.8)	2%	96.2 (86.8–99.5)	100 (92.6–100)	93.6 (83–97.1)	97.4 (87.1–99.1)	0.98 (0.95–1)
Subpleural positive	93 (86.1–97.1)	7%	96 (86.3–99.5)	90 (78.2–96.7)	95.7 (85.2–98.9)	90.6 (80.7–95.7)	0.93 (0.89–0.98)

For each variable, the performances are estimated on 100 randomly sampled patients with a prevalence of 50% regarding the feature of interest

All measurements are given with 95% confidence interval

*Abbreviations*: *AUROC* area under the ROC curve, *GGO* ground glass opacity, *NPV* negative predicted value, *PPV* positive predicted value

A total of 1721/49,311 (3.5%) reports were positive for GGOs, 263/49,311 (0.5%) were positive for reticulations and 1324/49,311 (2.7%) and 438/49,311 (0.9%) were positive for bilateral and subpleural lung abnormalities, respectively. Overall, 2539/49,311 (5.1%) reports exhibited one of these 4 features, 494/49,311 (1%) exhibited two features, 65/49,311 (0.1%) exhibited three features and 6/49,311 (0.01%) exhibited four features.

Combining GGOs and subpleural lung abnormalities resulted in 147/49,311 (0.3%) CT scans. One was excluded due to impossible image extraction, leading to 146 target CT scans. The characteristics of the entire population and the target CT scan subgroup are also shown in Table 1.

### Time-series analysis

The highest number of CT scans occurred on February 24, 2020 (*n* = 2031). The highest number of target CT scans occurred in the last week of the study (*n* = 34, 160, 49, and 14 observations, respectively). Supplementary Data S3 shows the raw time series.

**Fig. 5:**
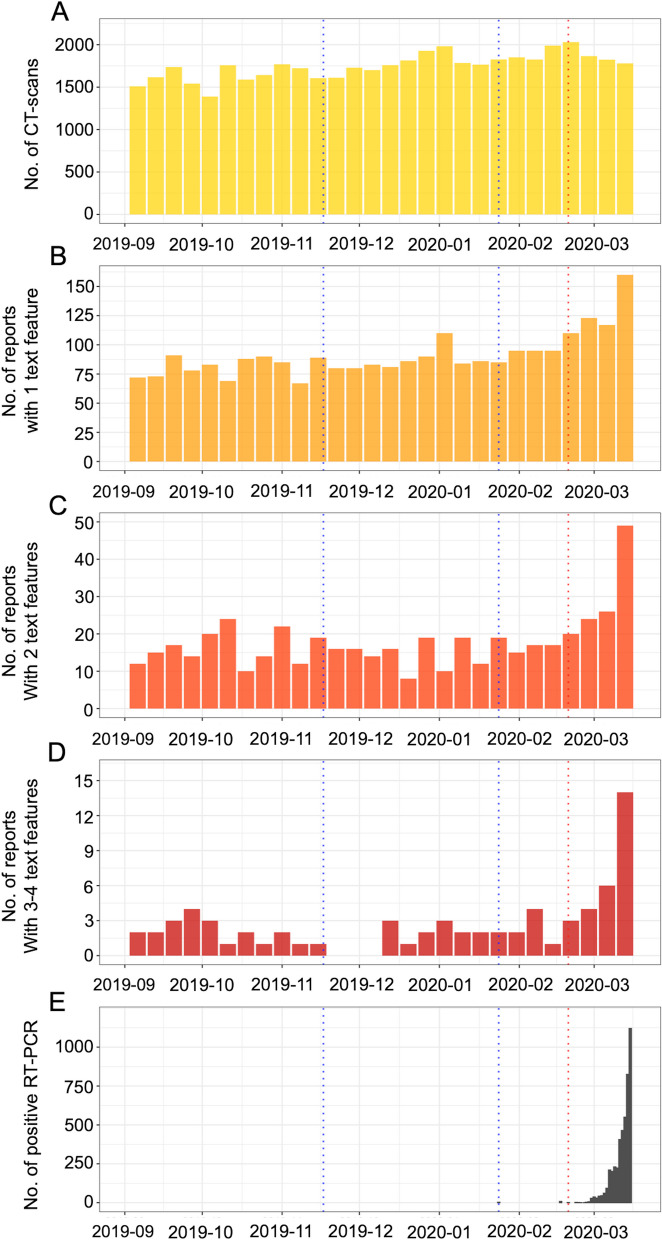
Time series analysis indexed by week. **A** Number (no.) of CT scans performed at IMADIS teleradiology during the study period. No. of reports with (**B**) 1, (**C**) 2, (**D**) 3 or 4 out of 4 text features identified by the text mining approach. **E** Daily number. of patients with a positive RT-PCR for COVID-19 according to French ‘*Santé Publique France*’. Red dotted line: February 17, 2020, i.e. break point for the presence of at least 3 radiological labels among ‘bilateral’, ‘supleural’, ‘ground glass opacities’ and ‘reticulations’. Blue dotted lines: first official Chinese patient (November 17, 2019) and first official patients in France (January 24, 2020)

Figure 5 represents the total number of reports and the number of reports with 1, 2 and ≥ 3 positive features in each week. There was a significant breakpoint on the week of February 17, 2020, for these last 3 variables (lowest BICs for this week = 257.1, 200.4 and 133.01) but not for the total number of reports. Indeed, the trends of the corresponding time series (Fig. 5B–D) were flat overall and then demonstrated a marked increase until the end of the inclusion period, which matched the marked increase in RT-PCR-positive cases recorded by SPF (Fig. 5E).

The comparisons of patients included from September 2019 to November 2019 against those included from December 2019 to February 2020 showed no significant results except for (1) patients’ age (*p* = 0.0098), with slightly older patient the second time period on average (57.4 ± 22.7 years vs. 57.9 ± 22.7 years), and for (2) presence of reticulations (*p* = 0.0307) with lower proportion of patients between December 2019 and February 2020 (103/24,018 [0.4%] vs. 122/21,154 [0.6%]) (Table 3).

**Table 3:** Comparisons of clinical and radiological characteristics in patients included from September 2019 to November 2019 against those included from December 2019 to February 2020

**Characteristics**	**From September 2019 to November 2019**	**From December 2019 to February 2020**	***p*-value**
**Age**			0.0098*
Mean ± SD (range)	57.4 ± 22.7 (0–99)	57.9 ± 22.7 (0–100)	
**Sex**			0.9287
Women	10,162/21,154 (48)	11,549/24,018 (48.1)	
Men	10,992/21,154 (52)	12,469/24,018 (51.9)	
**French regions initially affected by the outbreak**			0.1607
No	7594/21,154 (35.9)	8469/24,018 (35.3)	
Yes	13,560/21,154 (64.1)	15,549/24,018 (64.7)	
**GGO**			0.3987
Absent	20,488/21,154 (96.9)	23,227/24,018 (96.7)	
Present	666/21,154 (3.1)	791/24,018 (3.3)	
**Reticulations**			0.0307*
Absent	21,032/21,154 (99.4)	23,915/24,018 (99.6)	
Present	122/21,154 (0.6)	103/24,018 (0.4)	
**Subpleural abnormal finding**			0.8290
Absent	20,994/21,154 (99.2)	23,831/24,018 (99.2)	
Present	160/21,154 (0.8)	187/24,018 (0.8)	
**Bilateral abnormal finding**			0.6602
Absent	20,593/21,154 (97.3)	23,398/24,018 (97.4)	
Present	561/21,154 (2.7)	620/24,018 (2.6)	
**No. of positive text features**			0.6393
0 out of 4	19,898/21,154 (94.1)	22,583/24,018 (94)	
1 out of 4	1027/21,154 (4.9)	1199/24,018 (5)	
2 out of 4	206/21,154 (1)	209/24,018 (0.9)	
3 out of 4	22/21,154 (0.1)	24/24,018 (0.1)	
4 out of 4	1/21154 (0)	3/24,018 (0)	

^*^p < 0.05—tests are chi-square tests except for patients’ age, which corresponded to unpaired Mann–Whitney test after verifying the lack of normality

Data are number of patients with percentage in parentheses, except for age

*Abbreviations*: *GGO* ground glass opacity, *no* number, *SD* standard deviation

### Radiological analysis of target patients

We confirmed the accuracy of the text mining approach by comparing the raw texts with the provided tags. In the 146 selected reports, we found no mistakes (i.e. accuracy = 100% for the four radiological features).

Second, we compared the initial radiological readings with the consensus of the 3 retrospective readings (Table 4): the agreement for GGOs was 98.6% (144/146); GGOs were present according to only 1 of the 3 radiologists for 2 patients (Kappa = 0.98, 95%CI = 0.96–1, *p* < 0.0001). However, in this subcohort, the concordance for reticulations was 60/146 (41.1%) (Kappa = 0.256, 95%CI = 0.104–0.399, *p* = 0.0008).

**Table 4:** Radiological features, algorithm predictions and inter-rater agreement in patients with ‘target reports’ (*n* = 146)

**Characteristics**	**Patients**^**a**^	**Inter-rater agreement**^**b**^
**SFR-like classification**^**c**^		0.656 (0.601–0.717)
V-V-V	22/146 (15.1%)	
V-V-IV	3/146 (2.1%)	
V-IV-IV	9/146 (6.2%)	
IV-IV-IV	3/146 (2.1%)	
Others	109/146 (74.6%)	
**CART predicted probabilities**	0.739 ± 0.141	0.288 (0.2–0.39)
**Step-LR predicted probabilities**	0.622 ± 0.243	0.532 (0.464–0.597)
**GGO**	144/146 (98.6%)	0.904 (0.885–0.923)
**GGO predominant pattern**	110/146 (75.3%)	0.367 (0.263–0.46)
**Intralobular reticulations**	60/146 (41.1%)	0.33 (0.243–0.417)
**Subpleural predominant pattern**	105/146 (71.9%)	0.399 (0.311–0.487)
**bronchitis syndrome**	41/146 (28.1%)	0.275 (0.188–0.382)
**Diffuse lesions (≥ 2 lobes)**	100/146 (68.5%)	0.498 (0.402–0.589)
**Band-like condensations**	35/146 (24%)	0.366 (0.255–0.472)

*Abbreviations*: *CART* classification and regression trees, *GGO* ground glass opacities, *SFR* French ‘*Société Française de Radiologie’*, *Step-LR* stepwise logistic regression

^a^Data are number of patients (with percentage in parentheses), except for probabilities for positive RT-PCR according to the predictive models, which are mean probability ± standard deviation

^b^Inter-rater agreement is Krippendorff’s alpha with 95% confidence interval

^c^The findings for SFR-like classification correspond to the 3 readings by senior radiologists *X*_1_-*X*_2_-*X*_3_, where *X*_i_ ∈ {I, II, III, IV, V}, arranged in descending orders

Table 4 shows the prevalence, average predictions and inter-rater agreements of the radiological features. The inter-rater reproducibility of the SFR classification was high, with α_K_ = 0.656 (95%CI = 0.601–0.717). The binary variables with the highest and lowest inter-rater α_K_ were the presence of GGOs (α_K_ = 0.904, 95%CI = 0.885–0.923) and bronchitis syndrome (α_K_ = 0.275, 95%CI = 0.188–0.382), respectively. The inter-rater agreement of the predictions using the Step-LR model was higher than those using the CART model (α_K_ = 0.532 versus 0.288).

Overall, 37/146 (25.3%) patients were classified as SFR-IV by at least 3 radiologists (i.e. highly suspicious, ≥ SFR-IV-IV-IV), including 22/146 (15.1%) classified as SFR-V by 3 radiologists (i.e. SFR-V-V-V). Of them, 2/22 (9.1%) patients were imaged in 2019, 2/22 (9.1%) in January 2020, 4/22 (18.2%) in February 2020 and 14/22 (63.6%) in March 2020 (Table 5).

**Table 5:** Distribution of the target reports and highly suspicious chest CT scans (with corresponding predicted probabilities for positive RT-PCR) over the study period

	**Target reports** **(*n* = 146)**	**SFR ≥ IV-IV-IV** **(*n* = 37)**	**SFR = V-V-V** **(*n* = 22)**
**No. of patients per month**			
September 2019	17	**3**	2
October 2019	13	**2**	0
November 2019	11	**2**	0
December 2019	17	**0**	0
January 2020	24	**2**	2
February 2020	21	**6**	4
March 2020	43	22	14
**Average predicted probabilities**			
CART model	0.739 ± 0.141	0.831 ± 0.057	0.853 ± 0.038
Step-LR model	0.622 ± 0.243	0.852 ± 0.118	0.926 ± 0.045

Data are number of patients (with percentage in parentheses), except for probabilities for positive RT-PCR according to the predictive models, which are mean probability ± standard deviation

Numbers in bold correspond to the patients who were included in the clinical and biological complementary investigations

*Abbreviations*: *CART* classification and regression trees, *GGO* ground glass opacities, *SFR* French ‘*Société Française de Radiologie’*, *Step-LR* stepwise logistic regression

The Step-LR predicted probabilities were significantly higher in the SFR-V-V-V patients versus other comparisons (0.926 ± 0.045 versus 0.568 ± 0.223, Mann–Whitney *p* < 0.0001) and in the patients with an SFR of at least IV by 3 radiologists versus others (0.852 ± 0.118 versus 0.544 ± 0.225, Mann–Whitney *p* < 0.0001). Similar significant results with smaller differences between groups were found with the CART model (both *p*-values < 0.0001, with predicted probabilities of 0.853 ± 0.038 for SFR V-V-V, 0.831 ± 0.057 for SFR ≥ IV-IV-V and 0.708 ± 0.148 for SFR < IV-IV-IV) (Table 5).

When proposed, the alternative diagnoses in 109 remaining patients (i.e. < SFR-IV-IV-IV) were other infectious (broncho) pneumonitis (*n* = 27), interstitial diseases (*n* = 12), contusions (*n* = 11), lung infarcts (*n* = 11), pulmonary oedema (*n* = 5) and ventilatory disorders (*n* = 5), with 38 discordant proposals.

### Final diagnoses in patients with high suspicion of COVID-19 on CT scan before the official beginning of the pandemic

**Fig. 6:**
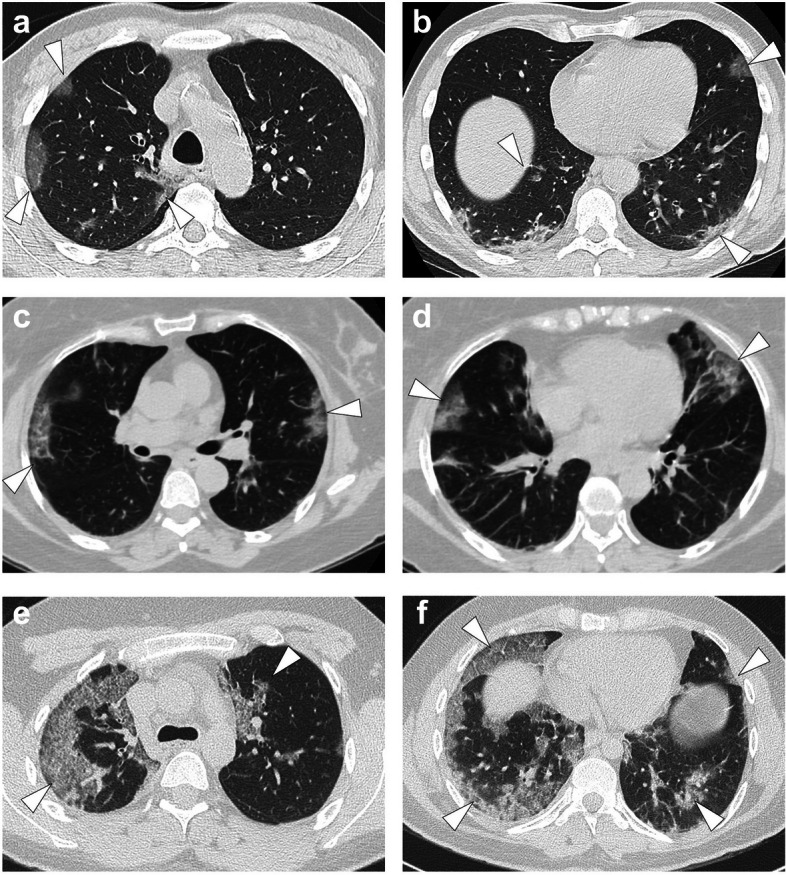
Differential diagnoses identified with the patients’ level analysis before February 2020. **a**, **b** Fifty-one years old man with kidney transplant addressed to the emergency for couch, dyspnoea and inflammatory syndrome in September, 2019. Final diagnosis was influenza A virus. **c**, **d** Fifty years old woman without significant medical history addressed to the emergency for flu syndrome, dyspnoea and low oxygen saturation (92%). Final diagnosis was influenza A virus. **e**, **f** Twenty-six years old male with dyspnoea, cough, inflammatory syndrome and low oxygen saturation (93.5%), already treated with 4 days of amoxicillin. Final diagnosis was influenza A virus and Mycoplasma pneumonia co-infection. All CT images are in axial plan and lung kernel. They showed bilateral subpleural ground glass opacities (white arrowheads) possibly combined with intralobular reticulations (providing a crazy paving pattern) and band-like condensations

Four patients were identified with typical COVID-19 chest CTs according to expert readings. In one patient (date of chest CT: January 25, 2020), no biological sample was available, and no infection was identified. Of the 3 others, 2 patients were positive for influenza A, and one was positive for both influenza A (using RT-PCR) and mycoplasma pneumonia (IgM +). Their chest CTs are displayed in Fig. 6.

## Discussion

We revisited the very early phase of COVID-19 outbreak in French emergency departments to investigate whether there was evidence for early circulation of SARS-CoV-2 before the ‘official’ start of the pandemic. We developed an original two-step complementary approach relying on both radiological reports and images. We showed that at both the macroscopic (population) level and patient level, there was no evidence that SARS-CoV-2 was circulating in the territories serviced by the included centres before the official pandemic started.

Using text mining of radiological reports of a large cohort of 49,311 chest CTs from consecutive patients referred to emergency departments, our dictionary and rule-based method enabled us to label the reports for the presence of four features, GGOs, subpleural abnormalities, bilateral abnormalities and reticulations, which were systematically quoted in the definition of a typical COVID-19 chest CT in the national guidelines [19]. We purposely investigated CT scans covering entirely or partly the chest and not radiographs in order to increase our ability to capture radiological reports with features suggestive of COVID-19 lung disease. Indeed, radiographs are known to be less sensitive than chest CT to detect COVID-19 lung disease (sensitivity of 56% according to Borakati et al., with 98% of chest radiographs being normal in asymptomatic or minimally symptomatic patient according to Kuo et al.) [20, 21]. Moreover, although COVID-19 lung disease is known to predominate in the lower lobes, typical lesions are also frequent in the right upper lobe, right middle lobe and left upper lobe (about 65%, 55% and 70% of patients with proven infection according to Bao et al.) [22]. Next, we plotted the number of reports with at least one, two, three and four features against time and analysed the time series concomitantly with the epidemiological time series provided by French Public Health [5]. Visual analysis showed a temporal alignment of patients with at least three and four features (named ‘radiological time series’) with the epidemiological time series but no anticipation of the epidemiological time series by the radiological time series. In addition, we identified a structural breakpoint on February 17, 2020, but not before, which highlights that no significant changes in the radiological time series occurred before February 17, 2020. These results were confirmed by a second approach in which we compared the clinical and text features in patients included from September 2019 to November 2019 (i.e. before the 1st reported patient with SARS-CoV-2) against patients included from December 2019 to February 2020. Patients were slightly older in this second time period, which is not surprising as it corresponds to winter in France with higher rates of visits to emergency departments and hospitalisation for elderly. Additionally, the presence of reticulations was significantly lower in the second time period. It must be noted that a majority of partner centres (68.9%) and examinations (64.5%) sampled the French regions where the pandemic was known to start [5].

This first part of our method also enabled us to automatically identify a subset of 146 patients with bilateral abnormalities and GGOs. As these features lacked specificity for COVID-19 infection, the CT scans were reviewed by 3 expert radiologists according to the SFR-SIT system, and we only included the 22 patients with a typical CT as scored by 3 out of 3 radiologists. It must be noted that ‘typical findings for COVID-19’ according to the SFR-SIT demonstrates similar diagnostic performances (i.e. sensitivity = 75% and specificity of 92%) as the highest category of COVID-19 reporting and data systems (CORADS) (i.e. sensitivity = 67% and specificity of 91.3% in the meta-analysis by Islam et al.) [9, 23]. To strengthen our suspicion, we applied a previously published machine-learning model, which provided an average probability of positive RT-PCR of 92.6% [16]. Four of these patients presented before February 2020 (2 in December 2019 and 2 in January 2020). For the 3 analysable patients, a diagnosis of influenza A infection (with a concomitant Mycoplasma pneumonia infection) was confirmed. The fourth patient remained without an identified infectious agent. However, his diagnosis (on January 25, 2020) occurred 1 day after the 1st official COVID-19 patient. It must be noted that the purpose of our work was not to develop new machine-learning models to predict positive RT-PCR for SARS-CoV-2 (as it would have required to obtain the RT-PCR status for all included patients) but to re-use previously published models that are easy to apply on new data with good diagnostic performances in order to reinforce the suspicion of COVID-19 of the target reports.

Our study emphasises the value of common information tools as employed in teleradiological structures working for several partners throughout a wide territory because they can rapidly provide databases that can be analysed at different scales: (i) macroscopically, to identify trends and breakdowns in radiological activities and findings [8], and (ii) microscopically, to identify small groups of patients for specific biological analyses. We believe that this two-steps approach, which combines (i) text mining to automatically annotate radiological reports for pathological radiological features in large databases and (ii) time series analysis to detect abnormal trends in the use of those radiological features over time, could be helpful for detecting and monitoring emerging and recurrent infectious diseases.

At both levels, the conclusions were similar: there is no evidence that SARS-CoV-2 was already actively circulating in the areas covered by our partner hospitals. This finding disagrees with the studies by Carrat et al. and Apolone et al. [7, 24, 25]. They performed an enzyme-linked immunosorbent assay (ELISA) test to detect anti-SARS-CoV-2 IgG antibodies in patients included in the CONSTANCES cohort (a general population-based cohort) [26], which were complemented by neutralising antibody testing. Carrat et al. found that 353 out of 9144 French adults (3.9%) had a positive IgG test against SARS-CoV-2, including 13 patients with confirmation based on neutralising antibody testing, between November 2019 and January 2020 [7]. However, the findings have been debated from a statistical point of view by Samuel et al. and may also be due to ELISA false-positives, such as common cold coronavirus, and confidence intervals around the results [27].

In addition to other infectious pneumonitis, differential diagnoses for COVID-19 were not encountered in our cohort, such as cryptogenic organising pneumonitis, pulmonary alveolar proteinosis or acute exacerbation of pulmonary fibrosis [28, 29]. This could be explained by the fact that those patients are generally primarily referred to specialised imaging departments and not to emergency departments.

Finally, this study stressed the lower performance of diagnostic tools developed for suspected SARS-CoV-2 infection when prevalence is low [9, 30]. According to Ohana et al., although the overall accuracy was preserved regardless of the ascending, peak and descending period of the 1st pandemic wave, the PPV appeared strongly affected by prevalence (i.e. PPV = 76% during the ascending period, for a prevalence of 37%, versus PPV = 90.8% during the peak period, for a prevalence of 64%).

Our study has limitations. First, although it involved 61 emergency departments throughout France, our cohort did not represent the entire French territory, and some regions were underrepresented. Additionally, although IMADIS is dedicated to emergency radiology during on-call period, we could not exclude that some patients were already hospitalised; however, their CT scans were always requested for an emergency condition and performed in an emergency setting. Second, biological samples were rarely available for complementary analysis. Indeed, before the beginning of the pandemic, there was no justification to keep blood or bronchial samples in biobanks. Third, knowledge of the radiological CT patterns was more superficial before the implementation of scientific publications, guidelines and educational materials following the beginning of the pandemic. Moreover, the radiologists who initially interpreted the imaging were not mandatorily experts in chest imaging, so they could have missed subtle thoracic radiological findings. This could lead to underestimation of the real number of affected patients. Fourth, the population-level approach relied on the analysis of the textual content of radiological reports and not directly on CT image, which could bias the real presence of the four radiological features of interest (especially if the radiologist who initially interpreted the imaging missed the feature). However, it would be hardly feasible to recruit a sufficient number of senior radiologists with expertise in chest imaging to review nearly 50,000 CTs. Future research could include deep learning models able to automatically detect interesting patterns in the chest CT image. However, false positive and false negative findings could still be possible and finding human resources to verify errors on thousands of observations will always be difficult. Fifth, more complex NLP models could have been investigated to automatically annotate the radiological reports, for instance text vectorisation followed by the training and validation of supervised machine-learning algorithms (such as random forests, support vector machine or naïve Bayes) or Bidirectional Encoder Representations from Transformers (BERT) [31]. However, since this task was simple (i.e. to detect the presence of a radiological feature with a few possibility to explain it verbally) and since we obtained good performances with rule-based models, training such models did not appear necessary.

## Conclusion

In summary, we proposed an innovative approach taking advantage of both text and imaging data stored in radiological databases to revisit a controversial issue, namely, the ‘real’ start of the COVID-19 pandemic in France. Using (1) a population-level approach combining text mining, time series analysis and an epidemiological dataset and (2) a patient-level approach with careful retrospective reading of chest CT scans complemented by analysis of samples performed contemporarily to the chest CT, we showed no evidence that SARS-CoV-2 was actively circulating in France before February 2020.

### Supplementary Information

**Additional file 1:** **Supplementary Data S1.** Principle of the two predictive models based on radiological features and used in the study, as developed by Shuster P, Crombé A et al. [14]. **Supplementary Data S2.** Types of CT scanners involved in the study. **Supplementary Data S3.** Weekly raw data for the time series analysis.

## Data Availability

Data is available on request from the corresponding author.

## Abbreviations

95%CI: 95% Confidence interval
BIC: Bayesian information criterion
CART: Classification and regression tree
CORADS: COVID-19 reporting and data systems
COVID-19: Coronavirus disease 2019
CT: Computed tomography
ELISA: Enzyme-linked immunoassay
GGO: Ground glass opacities
NPV: Negative predictive value
PPV: Positive predictive value
RSS: Residual sum of squares
RT-PCR: Reverse transcriptase polymerase chain reaction
s.d.: Standard deviation
SARS-COV-2: Severe acute respiratory syndrome coronavirus 2
SFR: French ‘*Société Française de Radiologie*’ [French Society of Radiology]
SPF: French ‘*Santé Publique France*’ [France Public Health]
Step-LR: Stepwise logistic regression
α_K_: Krippendorff’s alpha

## References

[1] Crombé A, Bensid L, Seux M (2023). Impact of vaccination and the omicron variant on COVID-19–related chest CT findings: a multicenter study. Radiology.

[2] Hammer MM (2023). The evolution of COVID-19: omicron and subvariants. Radiology.

[3] Lee JE, Hwang M, Kim Y-H (2023). Comparison of clinical outcomes and imaging features in hospitalized patients with SARS-CoV-2 Omicron subvariants. Radiology.

[4] CDC (2020) Coronavirus disease 2019 (COVID-19). In: Centers for Disease Control and Prevention. https://www.cdc.gov/coronavirus/2019-ncov/variants/variant-classifications.html. Accessed 31 Aug 2022

[5] Tableau de bord COVID-19. In: Gouvernement.fr. https://www.gouvernement.fr/info-coronavirus/carte-et-donnees. Accessed 31 Aug 2022

[6] Deslandes A, Berti V, Tandjaoui-Lambotte Y, et al (2020) SARS-CoV-2 was already spreading in France in late December 2019. International journal of antimicrobial agents 55:. 10.1016/j.ijantimicag.2020.106006 10.1016/j.ijantimicag.2020.106006 PMC7196402 32371096

[7] Carrat F, Figoni J, Henny J (2021). Evidence of early circulation of SARS-CoV-2 in France: findings from the population-based “CONSTANCES” cohort. Eur J Epidemiol.

[8] Crombé A, Lecomte J-C, Banaste N (2021). Emergency teleradiological activity is an epidemiological estimator and predictor of the COVID-19 pandemic in mainland France. Insights Imaging.

[9] Nivet H, Crombé A, Schuster P (2021). The accuracy of teleradiologists in diagnosing COVID-19 based on a French multicentric emergency cohort. Eur Radiol.

[10] Bai HX, Hsieh B, Xiong Z (2020). Performance of radiologists in differentiating COVID-19 from non-COVID-19 viral pneumonia at chest CT. Radiology.

[11] Kovács A, Palásti P, Veréb D (2021). The sensitivity and specificity of chest CT in the diagnosis of COVID-19. Eur Radiol.

[12] Salameh J-P, Leeflang MM, Hooft L (2020). Thoracic imaging tests for the diagnosis of COVID-19. Cochrane Database Syst Rev.

[13] Prokop M, van Everdingen W, van Rees Vellinga T, et al (2020) CO-RADS – a categorical CT assessment scheme for patients with suspected COVID-19: definition and evaluation. Radiology 0:201473. 10.1148/radiol.2020201473 10.1148/radiol.2020201473 PMC7233402 32339082

[14] Simpson S, Kay FU, Abbara S (2020). Radiological Society of North America expert consensus document on reporting chest CT findings related to COVID-19: endorsed by the Society of Thoracic Radiology, the American College of Radiology, and RSNA. Radiology.

[15] Qin L, Yang Y, Cao Q (2020). A predictive model and scoring system combining clinical and CT characteristics for the diagnosis of COVID-19. Eur Radiol.

[16] Schuster P, Crombé A, Nivet H (2021). Practical clinical and radiological models to diagnose COVID-19 based on a multicentric teleradiological emergency chest CT cohort. Sci Rep.

[17] Zeileis A, Leisch F, Hornik K, Kleiber C (2002). Strucchange: an R package for testing for structural change in linear regression models. J Stat Softw.

[18] Krippendorff K, Fleiss JL (1978). Reliability of binary attribute data. Biometrics.

[19] (2020) Compte-rendu TDM THORACIQUE IV-. In: SFR e-Bulletin. https://ebulletin.radiologie.fr/actualites-covid-19/compte-rendu-tdm-thoracique-iv. Accessed 2 May 2023

[20] Borakati A, Perera A, Johnson J, Sood T (2020). Diagnostic accuracy of X-ray versus CT in COVID-19: a propensity-matched database study. BMJ Open.

[21] Kuo BJ, Lai YK, Tan MLM, Goh X-YC (2021). Utility of screening chest radiographs in patients with asymptomatic or minimally symptomatic COVID-19 in Singapore. Radiology.

[22] Bao C, Liu X, Zhang H (2020). Coronavirus disease 2019 (COVID-19) CT findings: a systematic review and meta-analysis. J Am Coll Radiol.

[23] Islam N, Ebrahimzadeh S, Salameh J-P (2021). Thoracic imaging tests for the diagnosis of COVID-19. Cochrane Database Syst Rev.

[24] Montomoli E, Apolone G, Manenti A (2021). Timeline of SARS-CoV-2 Spread in Italy: results from an independent serological retesting. Viruses.

[25] Apolone G, Montomoli E, Manenti A (2021). Unexpected detection of SARS-CoV-2 antibodies in the prepandemic period in Italy. Tumori.

[26] Zins M, Goldberg M (2015). The French CONSTANCES population-based cohort: design, inclusion and follow-up. Eur J Epidemiol.

[27] Samuel ARe: Carrat, (2022). Evidence of early circulation of SARS-CoV-2 in France: findings from the population-based “CONSTANCES” cohort. Eur J Epidemiol.

[28] Mueller-Mang C, Grosse C, Schmid K (2007). What every radiologist should know about idiopathic interstitial pneumonias. Radiographics.

[29] Frazier JL, Batra S, Kapor S (2010). Stereotactic radiosurgery in the management of brain metastases: an institutional retrospective analysis of survival. Int J Radiat Oncol Biol Phys.

[30] Ohana M, Muller J, Severac F (2021). Temporal variations in the diagnostic performance of chest CT for COVID-19 depending on disease prevalence: Experience from North-Eastern France. Eur J Radiol.

[31] Devlin J, Chang M-W, Lee K, Toutanova K (2019) BERT: pre-training of deep bidirectional transformers for language understanding. North American Chapter of the Association for Computational Linguistics

