# Supplementary material for: Emergency department CT examinations demonstrate no evidence of early viral circulation at the start of the COVID-19 pandemic—a multicentre epidemiological study
Source: Insights Imaging. 2024 Jan 17;15:14. doi: 10.1186/s13244-023-01590-8 (PMC10792140; doi:10.1186/s13244-023-01590-8)
Supplement: Supplementary file 1 — Additional file 1: Supplementary Data S1. Principle of the two predictive models based on radiological features and used in the study, as developed by Shuster P, Crombé A et al. [14]. Supplementary Data S2. Types of CT scanners involved in the study. Supplementary Data S3. Weekly raw data for the time series analysis. [file 13244_2023_1590_MOESM1_ESM.pdf]

Emergency department CT examinations demonstrate no evidence of early viral circulation at the start of the COVID-19 pandemic – a multicentre epidemiological study

ELECTRONIC SUPPLEMENTARY MATERIAL

Supplementary Data S1. Principle of the two predictive models based on radiological features and used in the study, as developed by Shuster P, Crombé A et al. [14].

Stepwise logistic regression model (Step-LR) model

	Coefficients (βi)	Examples[§]		
		A	B	C
0. (Intercept)	-2.758126351	1	1	1
1. Presence of GGO	1.089578460	0	1	1
2. Fibrotic band	1.5310410593	1	0	1
3. GGO predominant pattern	1.5366549953	0	1	1
4. Subpleural predominant distribution	0.9053515712	0	1	1
5. Diffuse lesions	0.8414110324	0	0	1
6. Intralobular reticulations	0.7052466524	0	0	1
7. Bronchial wall thickening	-1.758954141	1	0	0
**Probability for a positive SARS-COV-2 RT-PCR[§§]**		4.8%	68.4%	97.9%

NOTES:

[§] Examples correspond to 6 distinct clinical cases. Each variable has 2 levels: "1" if the variable Xi is present (for instance fever), and "0" if the variable Xi is absent (for instance lack of fever).

[§§] The probability for RT-PCR+ are calculated as follows:

$$\text{P(RT-PCR+)} = {}^{1}/_{1 + \exp\left[-\left(\beta_0 + \sum_{i=1}^{7} \beta_i \times X_i\right)\right]}$$

* *P* < .05 ; ** *P* < .005 ; *** *P* < .001. Significant results are highlighted in bold.

Abbreviations: 95%CI: 95% confidence interval; GGO: ground glass opacity; OR: odds ratio.

CART model

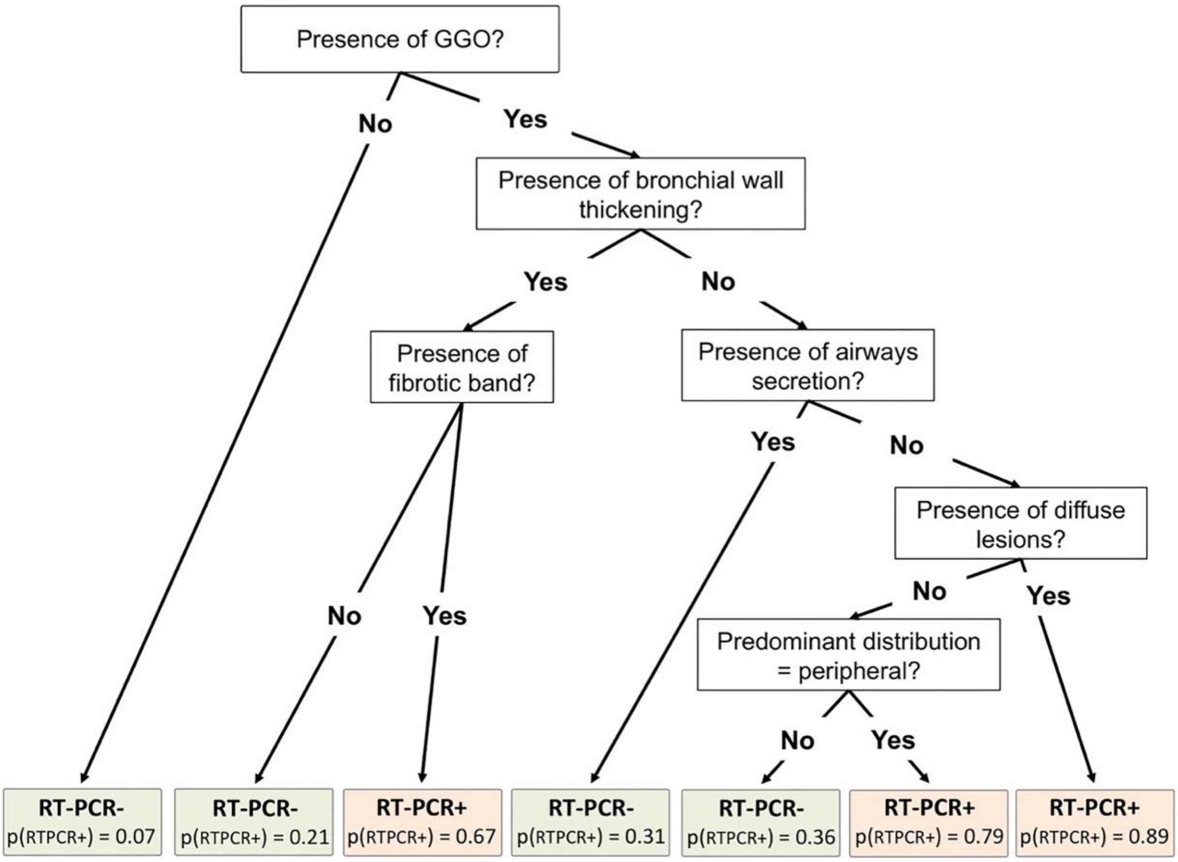

Presence of GGO?
No
Yes
Presence of bronchial wall thickening?
Yes
No
Presence of fibrotic band?
Presence of airways secretion?
Yes
No
Presence of diffuse lesions?
No
Yes
No
Yes
Predominant distribution = peripheral?
No
Yes
RT-PCR- p(RTPCR+) = 0.07
RT-PCR- p(RTPCR+) = 0.21
RT-PCR+ p(RTPCR+) = 0.67
RT-PCR- p(RTPCR+) = 0.31
RT-PCR- p(RTPCR+) = 0.36
RT-PCR+ p(RTPCR+) = 0.79
RT-PCR+ p(RTPCR+) = 0.89

Supplementary Data S2. Types of CT scanners involved in the study.

CT scanner name and manufacturer
Canon Aquilion Prime SP, Canon Medical
Canon Aquilion ONE, Canon Medical
Canon Aquilion Lightning, Canon Medical
GE Optima CT540, GE Healthcare
GE Optima CT660, GE Healthcare
GE Revolution EVO, GE Healthcare
GE Revolution Maxima, GE Healthcare
GE Revolution Ascend, GE Healthcare
GE Revolution Frontier, GE Healthcare
GE Discovery RT, GE Healthcare
GE Brightspeed 16B, GE Healthcare
GE BrightSpeed Elite CT, GE Healthcare
Philips Incisive CT, Philips Healthcare
Philips Ingenuity 64, Philips Healthcare
Philips Ingenuity 128, Philips Healthcare
Philips Ingenuity Core, Philips Healthcare
Siemens Somatom Definition AS 64, Siemens Healthineers
Siemens Somatom Definition AS 128, Siemens Healthineers
Siemens Somatom Definition Edge 128, Siemens Healthineers
Siemens Somatom Edge Plus, Siemens Healthineers
Siemens Somatom Flash, Siemens Healthineers
Siemens Fluorospot Compact FD, Siemens Healthineers
Siemens Somatom Go.Top, Siemens Healthineers
Siemens Somatom Perspective, Siemens Healthineers

SUPPLEMENTARY DATA S3. Weekly raw data for the time series analysis.

Week[§]	No. of patients	No. of reports with 1 text feature	No. of reports with 2 text features	No. of reports with 3 text features	No. of reports with 4 text features	No. of reports with 3 or 4 text features	No. of target reports
02/09/19	504	20	1	0	1	1	1
09/09/19	1510	72	12	2	0	2	4
16/09/19	1617	73	15	2	0	2	3
23/09/19	1737	91	17	3	0	3	5
30/09/19	1542	78	14	4	0	4	4
07/10/19	1389	83	20	3	0	3	2
14/10/19	1758	69	24	1	0	1	4
21/10/19	1589	88	10	2	0	2	2
28/10/19	1643	90	14	1	0	1	3
04/11/19	1769	85	22	2	0	2	3
11/11/19	1723	67	12	1	0	1	3
18/11/19	1606	89	19	1	0	1	3
25/11/19	1611	80	16	0	0	0	3
02/12/19	1730	80	16	0	0	0	1
09/12/19	1701	83	14	0	0	0	2
16/12/19	1759	81	16	2	1	3	4
23/12/19	1815	86	8	1	0	1	4
30/12/19	1928	90	19	1	1	2	5
06/01/20	1982	110	10	2	1	3	6
13/01/20	1785	84	19	2	0	2	7
20/01/20	1766	86	12	2	0	2	3
27/01/20	1827	85	19	2	0	2	8
03/02/20	1852	95	15	2	0	2	2
10/02/20	1826	95	17	4	0	4	6
17/02/20	1989	95	17	1	0	1	3
24/02/20	2031	110	20	3	0	3	7
02/03/20	1865	123	24	4	0	4	5
09/03/20	1824	117	26	5	1	6	10
16/03/20	1780	160	49	13	1	14	34

NOTES:

Abbreviation: No.: number.

[§] By convention, weeks start on the Tuesday and end on the next Monday. The numbers are indexed on the last day of the week (i.e., on Mondays).
